# Serum concentration of cobalt, molybdenum and zinc in aerobic, anaerobic and aerobic-anaerobic sportsmen

**DOI:** 10.1186/s12970-018-0233-z

**Published:** 2018-06-13

**Authors:** Marcos Maynar, Francisco Llerena, Francisco Javier Grijota, Mario Pérez-Quintero, Ignacio Bartolomé, Javier Alves, María Concepción Robles, Diego Muñoz

**Affiliations:** 1 Sport Sciences Faculty, University of Extremadura, Avenida de la Universidad s/n 10003, Cáceres, Spain; 2 School of Medicine, University of Extremadura, Avda. de Elvas s/n, 06071 Badajoz, Spain; 3 Education Faculty, University of Extremadura, Avenida de la Universidad s/n 10003, Cáceres, Spain; 4 Education Faculty, University of Salamanca, Henry Collet, 52-70, 37007 Salamanca, Spain

**Keywords:** Essential metals, Serum, Blood, Metabolism, Exercise, Adaptations

## Abstract

**Background:**

The aim of the present study was to determine changes in the serum concentrations of trace elements Cobalt (Co), Molybdenum (Mo) and Zinc (Zn) among high-level sportsmen.

**Methods:**

Eighty professional athletes of different metabolic modalities (aerobic, anaerobic and aerobic-anaerobic), were recruited before the beginning of their training seasons. Thirty-one sedentary participants of the same geographic area constituted the control group. Co, Mo and Zn analysis was performed by Inductively Coupled Plasma Mass Spectrometry (ICP-MS).

**Results:**

Serum concentration of Mo (*p* < 0.001) was higher among sportsmen compared to the control group values. Separated by modalities, the concentrations of Co in the aerobic-anaerobic athletes were lower (*p* < 0.01) than in the control group as well as than in the other athletes. The highest Mo concentration was found in anaerobic sportsmen (*p* < 0.001), followed by aerobic-anaerobic (*p* < 0.001) being both statistically higher in comparison with the control group. In relation to Zn, it was observed that aerobic-anaerobic (*p* < 0.001) and anaerobic (*p* < 0.001) sportsmen showed higher concentrations than the control participants. However, aerobic sportsmen showed lower concentrations (*p* < 0.01) than controls.

**Conclusion:**

This data manifest that long-term, daily physical training may induce variations in serum concentrations of several essential elements among sportsmen in comparison to untrained men and that these changes seems to be related to the sports modality practiced.

## Background

Essential trace elements such as cobalt (Co), molybdenum (Mo) and zinc (Zn) develop necessary roles in different physiological functions. Minimum alterations in its concentrations may induce critical alterations in its physiological functions as well as in the physical performance. An interesting point for physicians and sportsmen is that several of these minerals develop important functions in the biological processes of adaptation to exercise.

Co, Mo and Zn play essential roles in different adaptive processes to physical training. In this sense, Co enters in the composition of cobalamin and increases erythropoiesis [1]. Furthermore, Co is one of the microelements which influence the vascular system [2], dilating the vessels and inducing a hypotensive effect [3].

Among humans, Mo functions as an enzymatic cofactor of three enzymes (aldehyde oxidase, sulfite oxidase, xanthine oxidase dehydrogenase), which catalyze the hydroxylation of several substrates. Xanthine dehydrogenase catalyzes the transformation of hypoxanthine to xanthine and of xanthine to uric acid [4].

In relation to Zn, this elements inhibits nicotinamide adenine dinucleotide phosphate (NADPH) oxidation, also constitute an integral part of several metallothioneins as well as of Cu-Zn-Superoxide Dismutase (Zn-Cu-SOD) [5]. Furthermore, Zn develops an important anti-inflammatory response reducing the cytokines production [6]. Additionally, it has been reported that low blood Zn concentrations are linked to higher lactate production as well as lower glycemic and hepatic glycogen levels during physical efforts [7].

The metabolism of trace elements can be affected by many factors, including aging and exercise [8]. Physical activity induces many metabolic changes in the body as well as physiological adaptations. Regular physical training may increase essential trace element requirements, either by increases in the degradation rates or by decreasing their losses from the body. In this sense, it has been recently reported that physical training induces changes in serum concentrations of several biological minerals among sportsmen [9]. So, the aim of this study was to determine differences in serum Co, Mo, and Zn values in sportsmen in comparison to sedentary population as well as to evaluate if different metabolic exercise modalities induced differences in these serum minerals concentrations.

## Methods

This manuscript constitutes the continuation of Maynar et al. [10], so, the methodology, techniques and protocols described below follow the same guidelines and procedures.

### Participants

This research was carried out under the Helsinki Declaration ethic guidelines, updated at the World Medical Assembly in Seoul in 2008, for research with human participants. All the participants were previously informed about the purpose of the study and gave their voluntary signed informed consent.

Eighty male professional athletes and 31 sedentary males participated in the present survey. All of them were living in Cáceres (Spain) and had been living in the same region for, at least, 2 years. The athletes had trained regularly for the two previous years. All participants completed a nutritional questionnaire about their nutritional habits to ensure they were not taking any vitamins, minerals or other supplementation and to guarantee they all had a similar diet.

The athletes were classified into four groups: athletes group (SPG) (athletes; *n* = 80) of all the modalities (aerobic + anaerobic + aerobic-anaerobic) with an average age of 20.32 ± 3.24 years; aerobic athletes group (AEG) (Aerobic: long distance runners; *n* = 28) average aged of 21.58 ± 4.39 years; anaerobic athletes group (ANEG) (Anaerobic: judo and speed athletes; *n* = 24) with an average age of 20.11 ± 2.56 years; and aero-anaerobic athletes group (AE-ANEG) (Aerobic-anaerobic: professional football players; 28 individuals) with and average age of 22.23 ± 4.30. Sedentary students, with an average age of 21.78 ± 3.52 years, formed the control group (CG).

The beginning of the survey started at the end of October coinciding with the beginning of their respective training seasons. All SPG participants had been performing high level physical training, at least, 2 years before the beginning of the survey. AEG training routine consisted of 120 Km/week, performing 3–4 days/week in aerobic conditions or aerobic fartlek and 2–3 days/week of intense series and interval training. The training routine of the ANEG consisted of 5–6 training days/week, performing 3–4 h/day of specific routines. AE-ANEG participants started their competition in September. All AE-ANEG sportsmen performed an average of 3–4 h/day, 4–5 days/week of mixed physical (aerobic-anaerobic intensities), technical and tactical exercises.

### Anthropometric measurements

The morphological characteristics of the participants were measured in the morning and always at the same time. Body height was measured to the nearest 0.1 cm using a wall mounted stadiometer (Seca 220. Hamburg. Germany), and body weight was measured to the nearest 0.01 kg using calibrated electronic digital scales (Seca 769. Hamburg. Germany) in nude, barefoot conditions. Body fat content was estimated from sum of 4 skinfolds (abdominal, suprailiac, tricipital and subescapularis) and sum of 6 skinfolds (previous skinfolds, thigh and calf skinfolds). The ∑4 and ∑6 skinfold thicknesses were measured with a Harpenden calliper (Holtain Skinfold Caliper. Crosswell, UK). Measurements were made by the same operator, skilled in kinanthropometry techniques, in accordance with the International Society for the Advancement of Kinanthropometry recommendations. Heart rate and blood pressure were determined using an automatic sphygmomanometer (Omron HEM-780. Osaka. Japan) by a skilled technician, and after a 5-min rest period in supine position.

### Physical performance evaluation

To evaluate the performance variables of the athletes and controls, an exercise test was performed in a treadmill (Powerjoc. Uk), equipped with a gas analyser (Metamax. Cortex Biophysik. Gmbh. Germany) and a Polar pulsometer (Polar. Norway). To ensure a warm-up phase, all participants ran before the test during 15 min ending at the initial speed of the test. For the test, the participants ran on a treadmill, incrementally in stages, until exhaustion, starting at a speed of 6 km·h-1 the CG and at 10 km·h-1 the SPG, increasing the speed at 1 km·h-1 every 400 m, with a stable slope of 1%, and always within the recommended parameters [11]. The 2 days before the test, training intensity was reduced, applying regenerative load in order to avoid pre-test fatigue.

The criteria used to determine maximal oxygen uptake (VO_2max_) was a stabilisation in oxygen uptake (VO_2_) accompanied of an increment in carbon dioxide (CO_2_) elimination, in respiratory exchange ratio (RER), and ventilatory volume (VE) induced by increments in exercise intensity (Km/h) in treadmill. To ensure that physical tests were until exhaustion, athletes should come to the test in recovered conditions (normal basal parameters) and the RER had to exceed 1.

To verify the theoretical differences among sport groups and to ensure that each participant was properly classified in each group, apart from maximal oxygen consumption, maximal RER (RER_max_) was used. The justification of this criteria is based in the concept of RER, which defines de ratio between oxygen (O_2_) consumed and carbon dioxide (CO_2_) eliminated. When RER exceeds 1.0 it means that the metabolic production of CO_2_ overmatches the O_2_ metabolic utilization, which implies that the exceed in CO_2_ elimination comes from anaerobic metabolisms. This idea has been demonstrated by Goedeckeet al [12]. These authors demonstrated that RER is highly determined by lactate production. In this sense, anaerobic sportsmen can produce, buffer and tolerate more lactate than aerobic ones [13] which is reflected in higher RER_max_ values. Furthermore, aerobic sportsmen are highly affected by lactate accumulation, which supposes one of the main muscle fatigue precursors and one of the main performance limiting factors [14]. The inability of aerobic sportsmen to buffer and tolerate lactate is reflected in their RER_max_ values, which should be virtually lower than anaerobic sportsmen values.

### Nutritional evaluation

In order to ensure participants were not taking any vitamins, minerals or other supplementations and in order to guarantee they were following a similar diet, all participants completed a dietary questionnaire. The questionnaire consisted in a 3-day daily nutritional record, in two pre-assigned weekdays and in 1 weekend day. In each day participants indicated, individually, the type, frequency and quantity (in grams) of every food consumed, then the nutritional composition of their diets was evaluated using different food tables and databases [15–17]. Results of this nutritional evaluation are collected in Tables 3 and 4.

### Blood samples collection

Participants were recruited at 9 am for the extraction of 5 mL of venous blood from the antecubital vein, using a plastic syringe fitted with a stainless-steel needle. All extractions were carried out in fasting conditions. In order to obtain reliable results, all athletes were advised to rest the two previous days before the tests as well as not to training the morning before the extractions. The blood sample was collected into a metal-free polypropylene tube (previously washed with diluted nitric acid). Later, the blood sample was centrifuged at 2500 rpm for 10 min at room temperature to isolate the serum. The serum was aliquoted into an Eppendorf tube (previously washed with diluted nitric acid) and conserved at − 80 °C until the biochemical analysis.

### Experimental protocol

#### Serum trace elements determination

Co, Mo and Zn was performed by inductively coupled plasma mass spectrometry (ICP-MS). Decomposition of the organic matrix was performed by heating for 10 h at 90 °C after the addition of 0.8 mL HNO_3_ and 0.4 mL H_2_O_2_ to 1 mL of serum [18]. The samples were then dried at 200 °C on a hot plate. Sample reconstitution was carried out by adding 0.5 mL of nitric acid, 10 μL of In (10 mg·L-1) as internal standard and ultrapure water to complete 10 mL. Reagent blanks, element standards and certified reference material (Seronorm, lot 0511545, Sero AS Billingstand, Norway) were prepared in the same way and used for accuracy testing. Before the analysis, the commercial control materials were diluted according to the recommendation of the manufacturer. Digested solutions were assayed by an ICP-MS Nexion model 300D (PerkinElmer, Inc., Shelton, CT, USA) equipped with a triple quadrupole mass detector in addition to a reaction cell/collision that allows operation in three modes: STD (no reaction gas), KED or kinetic energy discrimination (with helium as the collision gas) and DRC or reaction mode (with ammonia as the reaction gas). Both collision and reaction gases such as argon plasma had a purity of 99.999% and were supplied by Praxair (Madrid, Spain). Two mass flow controllers regulated gas flows. The frequency generator was free-swinging and worked at 40 Mhz. Three replicates were analysed per sample. Quantification was performed by indium (In) as internal standard. The values of the standard materials of each element (10 μg·L-1) measured for quality controls were in good agreement with intra and inter-assay variation coefficients of less than 5%.

#### Statistical evaluations

Statistical analyses were performed with the *IBM SPSS Statistic* software version 19 for *Windows*. The results are expressed as *x* ± *s*, where *x* are the mean values and *s* the standard deviation. A *p*-value of p ≤0.05 was considered statistically significant.

The normal distribution of the variables was assessed using the Shapiro-Wilks test. To evaluate differences between CG and SPG Student test was used. One-way Anova test followed by Bonferroni post hoc test was used to determine differences between control and sportsmen groups. The differences were evaluated in the anthropometric, cardiorespiratory, nutritional and seric variables. A p-value < 0.05 was considered statistically significant.

## Results

The results are separated in two sections and in two different tables for each section, in the firsts we will compare the results obtained among all the athletes with the control group; in the second tables of each section results are compared among the athletes separated by the metabolic type of activity with the control group and among themselves.

### Anthropometric and cardiorespiratory characteristics

Table 1 shows data obtained in the anthropometric and cardiorespiratory evaluations from the two groups. It can be observed that SPG had total body, body mass index (BMI), weight and fat values (Σ of skinfolds) significantly lower (*p* < 0.001) than the CG. VO_2max_ was significantly higher (*p* < 0.001) in athletes compared to CG. Resting heart rate was lower (*p* < 0.01) in athletes in comparison to the CG. As it can be observed in this survey the different groups are well-characterized.

**Table 1:** Anthropometric characteristics of control group and athletes

	CG (*n* = 31)	SPG (*n* = 80)
Height (m)	1.76 ± 0.057	1.76 ± 0.07
Weight (kg)	78.21 ± 12.19	65.31 ± 7.55^***^
Body Mass Index	23.81 ± 3.14	20.25 ± 1.73^***^
Age (years)	21.78 ± 3.52	20.32 ± 3.24
Σ4 skinfolds (mm)	52.02 ± 23.77	35.12 ± 9.29^***^
Σ6 skinfolds (mm)	88.82 ± 34.50	45.85 ± 16.69^***^
VO_2Máx_ (mL·min^− 1^·kg^− 1^)	41.89 ± 7.50	64.08 ± 5.27^***^
HR _basal_ (beat·min^− 1^)	71.58 ± 7.38	59.75 ± 9.66^*^

Student test (^*^p < 0.05; ^**^p < 0.01; ^***^p < 0.001) differences between control and sportsmen groups

Table 2 shows data about the participants of the SPG separated by metabolic exercise specialty. AEG and ANEG show the lowest (*p* < 0.001) body weight, followed by AE-ANEG (*p* < 0.05). However, in the case of body fat, AEG have the least (*p* < 0.001) values followed by AEG (*p* < 0.001) and AE-ANEG (*p* < 0.01). Concerning the BMI, ANEG showed the lowest values (*p* < 0.001) in comparison to CG, followed by AEG (*p* < 0.001) and AE-ANEG (*p* < 0.001), being the values of the last group statistically higher (*p* < 0.001) than the values of the AEG and ANEG. In relation to the oxygen update, the AEG have the highest (*p* < 0.001) value followed by ANEG (*p* < 0.001) and AE-ANEG (*p* < 0.01) in comparison to the CG. For its part, resting heart rate was lower (*p* < 0.01) in AEG followed by AE-ANEG (*p* < 0.05) and ANG (*p* < 0.05).

**Table 2:** Characteristics of control group and sportsmen classified by metabolic modalities

	CG (*n* = 31)	AEG (*n* = 28)	ANEG (*n* = 24)	AE-ANEG (*n* = 28)
Height (m)	1.76 ± 0.05	1.77 ± 0.05	1.73 ± 0.07	1.80 ± 0.05
Weight (kg)	78.21 ± 12.19	64.95 ± 7.10^***^	64.91 ± 8.46^***^	73.78 ± 6.12^*†††■■■^
Body Mass Index	23.81 ± 3.14	21.01 ± 1.67^***^	20.73 ± 1.84^***^	22.71 ± 2.14^*†††■■■^
Age (years)	21.78 ± 3.52	21.58 ± 4.39	20.11 ± 2.56	22.23 ± 4.30
Σ4 skinfolds (mm)	52.02 ± 23.77	32.56 ± 8.75^***^	33.66 ± 9.87^***^	38.25 ± 10.06^**†^
Σ6 skinfolds (mm)	88.82 ± 34.50	49.69 ± 14.84^***^	56.32 ± 16.65^**††^	59.49 ± 17.10^**†††^
VO_2Máx_ (mL·min^− 1^·kg^− 1^)	41.89 ± 7.50	66.17 ± 8.36^***^	61.23 ± 3.00^***†^	59.85 ± 4.53^**††■^
RER_max_	1.17 ± 0.10	1.10 ± 0.08^*^	1.25 ± 0.06^*^	1.15 ± 0.09
HR_basal_ (beats·min^− 1^)	72.58 ± 7.38	54.90 ± 10.85^**^	62.85 ± 7.57^*††^	57.21 ± 10.51^*■^
HRmax (beats·min^− 1^)	184.32 ± 12.71	173.44 ± 8.3^*^	195.10 ± 8.45^■^	174.62 ± 11.68

Anova and Bonferroni tests. (^*^p < 0.05; ^**^p < 0.01; ^***^p < 0.001) differences between control group and aerobic, anaerobic and aerobic-anaerobic groups; (^†^*p* < 0.05; ^††^*p* < 0.01; ^†††^*p* < 0.001) differences between aerobic group and anaerobic and aerobic-anaerobic groups; (^■^*p* < 0.05; ^**■■■**^*p* < 0.001) differences between anaerobic group and aerobic-anaerobic group

### Nutritional intake of minerals

The information about the dietary intake of minerals among the participants is presented in Tables 3 and 4. As it can be observed in Table 3 sportsmen ingested higher values of daily Kilocalories (Kcal) than controls. Table 4 shows a significant difference between AEG and the other groups. No differences were found in the daily intake of the studied elements.

**Table 3:** Nutritional intake of trace elements with essential functions demonstrated: Co, Mo and Zn in control group and sportsmen

Elements (Reference Daily Intake)	CG (*n* = 31)	SPG (*n* = 80)
Energy (Kcal/d)	2243.64 ± 251.98	2860 ± 198.31^**^
Co (200–300 μg/d)	200.98 ± 186.16	220.71 ± 146.53
Mo (75–400 μg/d)	223.31 ± 184.33	262.66 ± 161.62
Zn (10–15 mg/d)	14.23 ± 6.54	12.99 ± 5.33

Student test (^**^p < 0.01) differences between control and sportsmen groups

**Table 4:** Nutritional intake of trace elements Co, Mo and Zn of control group and sportsmen separated by metabolic specialties

Elements (Reference Daily Intake)	CG (*n* = 31)	AEG (*n* = 28)	ANEG (*n* = 24)	AE-ANEG (*n* = 28)
Energy (Kcal/d)	2243.64 ± 251.98	2989.12 ± 200.22^***^	2215.22 ± 265.15	2571.13 ± 211.56
Co (μg/d)	200.98 ± 86.16	256.22 ± 77.16	225.15 ± 81.22	200.21 ± 63.22
Mo (μg/d)	223.31 ± 184.33	232.24 ± 156.12	212.33 ± 182.64	224.13 ± 171.51
Zn (Mg/d)	14.23 ± 6.54	12.85 ± 6.94	11.67 ± 9.21	12.62 ± 9,34

Anova and Bonferroni tests. (^***^ *p* < 0.001) differences between control group and aerobic, anaerobic and aerobic-anaerobic groups

### Serum mineral concentrations

Table 5 shows serum essential trace minerals values of CG and SPG. It is interesting that in the SPG exist higher basal concentrations of Mo and Zn, being statistically significant in the case of Mo (*p* < 0.001). Aversely, basal serum Co concentrations of SPG were lower than CG.

**Table 5:** Serum trace element concentrations of control group and high level sportsmen

Elements	CG (*n* = 31)	SPG (*n* = 80)
Co (μg/L)	0.823 ± 0.417	0.719 ± 0.089
Mo (μg/L)	0.339 ± 0.115	1.528 ± 0.886^***^
Zn (μg/L)	979.62 ± 114.48	1034.86 ± 316.23

Student test (^***^p < 0,001) differences between control sportsmen groups

Table 6 shows the serum concentrations of CG and of participants of the SPG separated by metabolic modalities. It can be observed in this table that Co serum concentrations in AEG and ANEG are similar to CG while AE-ANEG Co values are lower (*p* < 0.01) than CG and ANEG as well.

**Table 6:** Serum trace elements concentrations of control group and sportsmen classified by metabolic specialties

Elements	CG (*n* = 31)	AEG (*n* = 28)	ANEG (*n* = 24)	AE-ANEG (*n* = 28)
Co (μg/L)	0.823 ± 0.417	0.719 ± 0.131	0.749 ± 0.071	0.692 ± 0.059^■■**^
Mo (μg/L)	0.339 ± 0.115	0. 880 ± 0.822^***^	2.083 ± 0.674^***†††^	1.622 ± 0.711^***†††■^
Zn (μg/L)	979.620 ± 114.480	839.160 ± 242.160^**^	1183.52 ± 371.62^***†††^	1152.360 ± 146.870^***†††^

Anova and Bonferroni tests. (^**^p < 0,01; ^***^p < 0,001) differences between control group and aerobic, anaerobic and aerobic-anaerobic groups; (^†††^*p* < 0,001) differences between aerobic group and anaerobic and aerobic-anaerobic groups; (^■^*p* < 0,05; ^■■^*p* < 0,01) differences between anaerobic group and aerobic-anaerobic group

In relation to Mo, all sportsmen groups present higher (*p* < 0.001) concentrations than CG. The lowest concentrations were founded in AEG, with a significant difference (*p* < 0.001) between this and the other two groups. ANEG presented the highest serum concentrations, followed by AE-ANEG, with significant (*p* < 0.05) differences between them.

Regarding Zn significant differences between CG and the different exercise specialties was found; AEG concentrations were lower (*p* < 0.01) respect to CG, ANEG (*p* < 0.05) and AE-ANEG (*p* < 0.001) presented higher concentrations than AEG and CG. In the same way, the differences in AEG concentrations were significant lower (*p* < 0.001) in respect to ANEG and AE-ANEG.

## Discussion

The possible ergogenic role of minerals in human performance has been studied [19] as well as the effects of physical exercise on its body concentrations. In this research, it can be observed important differences in anthropometric variables as well as serum concentrations of essential trace metals between sportsmen and control (no sportsmen) participants. This data reinforces the differences found by Maynar et al. [10] and Maynar et al. [9] in their surveys, with similar subjects and in different elements.

As indicated in Tables 1 and 2, there were important anthropometric and fitness differences between groups. These tables show, as indicated by the total weight, the sum of the skinfolds, the maximum oxygen consumption and the resting heart rate, a clear adaptive response to exercise among sportsmen as consequence of long term, continuous training. If athletes are separated by metabolic specialties, those differences are maintained, being specific to the type of training that was followed in each specialty. In all cases the differences are clear, and this fact leads us to think that the exercise-induced effects could also affect the values of the minerals studied. This idea is based on the roles of these elements in the physiological processes linked to exercise, like the adaptations in the antioxidant enzymes [20].

An important fact in the metabolic behaviour of these elements, as well as its effect on physical performance and health, is the nutritional intake [21]. Despite similar nutritional intake of minerals, it can be observed significant differences in serum concentrations of essential trace metals between sportsmen and sedentary people. However, in all cases the mineral seric concentrations were within reference values of untrained, healthy participants, being the values, in μg/L, of each element: Co 0,21–7; Mo 0,5–2; Zn 700–1600 [22].

Thus, in relation to the Co, the values were similar among athletes of the different modalities as well as among CG participants. These results are in accordance with the obtained by Bergeret al. [23] in marathon runners.

In relation to Mo, data obtained in the present research could indicate a higher production of free radicals by exercise-induced ischemia-reperfusion in the muscles. This physiological phenomenon can be induced by high intensity muscle contractions [24], a typical situation among anaerobic physical efforts and, with minor magnitude, by long-duration aerobic exercise, this hypothesis is reinforced by the obtained data, being higher the Mo values among all sportsmen (of all modalities) than among CG.

Mo participates in oxide-reduction processes as an integral part of several enzymes like xanthine dehydrogenase, an enzyme which catalyzes the hypoxanthine transformation of xanthine to uric acid [4] which is considered an antioxidant substance. This biochemical process is augmented in sportsmen metabolism, especially among anaerobic exercises. In this survey the ANEG sportsmen presented the highest concentrations of seric Mo, followed by AE-ANEG participants and, finally, by AEG (*p* < 0.001). As it has been previously indicated, these differences in Mo values could be linked to a higher production of free radicals by ischemia-reperfusion in the SPG. Bergeret al [23], found similar results among marathon runners, but, in their case, without the reference of a control group. Augmented Mo concentrations would ease the formation of uric acid as well as decrease the damage caused by superoxide anions generated by xanthine oxidase in the ischemia-reperfusion processes, situation induced by high intensity muscular activities [24].

In this study, similar Zn concentrations were found in CG and SPG (Table 5). However, when athletes were divided according to the type of activity performed differences can be found between sportsmen and CG participants.

Zn is part of more than 300 enzymes involved in various functions of cellular metabolism, including metabolism of lipids, proteins and carbohydrates [25]. It is distributed in all organs, fluids and secretions of the human body.

In the study of the effect of physical training in plasma values of Zn, Rodríguez-Tuya et al. [26] found, as in the present survey, that plasma Zn concentrations were significant higher in anaerobic sportsmen than in control group. However, Rodríguez-Tuya et al. [26] found that aerobic group plasma Zn concentrations were significant higher than control group values whereas in the present research AEG serum Zn concentrations are lower than CG values.

As in the present survey, Michelettiet al. [27] detected the same situation among Italian athletes. They indicated that nutritional habits of elite athletes during training and competition are quite different from the standard diets of the majority of the population.

Endurance athletes often adopt an unusual diet in an attempt to enhance performance: an excessive increase of carbohydrates in their diets and a low intake of proteins and fat may lead to suboptimal Zn intake. The finding of De Carvalhoet al. [28] reinforces this idea, due to they found Zn deficiencies in serum of elite swimmers.

The low serum concentrations among AEG participants, may be due to an exercise-induced body Zn redistribution between body stores, bloodstream and tissues. Furthermore, the increased metabolism may lead to a deficiency of Zn, requiring supplementation in order to maintain normal values and high level performance [29, 30]. Furthermore, Andrade and Marreiro [31] found that during physical exercise, Zn compartmentalization may be impaired, which means that changes in the concentrations of this element may occur in different organic compartments, and this may reflect differences in blood values. However, the mechanisms involved in the metabolism of Zn, as well as its effects in the improvement of physical performance, are not fully understood yet. In all cases, decreases in Zn concentrations can lead to a reduction in the activity of a fundamental enzyme among endurance athletes, carbonic anhydrase [32] as well as to a decrease in the antioxidant defense system and, consequently, to an increase in oxidative stress in sportsmen cells [33]. For all these reasons, and as Karaet al. [34] indicated, Zn supplementation among athletes may beneficially contribute to their health and performance. However, it is striking that Zn concentrations were significantly (*p* < 0.001) higher in AE-ANEG and in ANEG (*p* < 0.05) than in CG and very significantly (*p* < 0.001) higher than in AEG. These data would be in relation with the data of found by Granell [35]. This suggest that changes of seric Zn can be linked to the type of exercise performed, being the higher values in cases of intense impacts in muscular tissues.

## Conclusions

This research manifests that essential minerals play a critical role in the normal metabolism in situations of continuous physical training. As a consequence of the predominant metabolism (aerobic or anaerobic) of physical training, serum concentrations of these elements suffer different changes specific of the nature of the efforts and training practiced.

These facts suggest a possible body redistribution of these elements and, although serum is a reference organic matrix, tissular or cellular samples are needed to know the real status of each mineral in the body. This constitute a limitation, because the possibility to get tissular samples among high level sportsmen is difficult, due to the disturbances and performance-limiting effects caused by invasive methods.

Finally, this survey suggest a different metabolic response in mineral metabolism induced by different kinds of exercise (aerobic, anaerobic or mixed) and further surveys are required to meet the optimal serum ranges of each element, the possible correlations between the mineral levels and the degree of physical performance and the specific daily demands for each modality.
